# Development of a Protein-Free Nucleic Acid Lateral Flow Assay for 

**DOI:** 10.1021/acs.analchem.5c01006

**Published:** 2025-07-08

**Authors:** Christine Aubrey C. Justo, Miriam Jauset-Rubio, Vasso Skouridou, Piet Cools, Lisa Himschoot, Abel Abera Negash, Guy Mulinganya Mulumeoderhwa, Alexandra Ibáñez-Escribano, Ciara K. O′Sullivan

**Affiliations:** 1 Interfibio Consolidated Research Group, Departament d’Enginyeria Química, 16777 Universitat Rovira i Virgili, Tarragona 43007, Spain; 2 Department of Diagnostic Sciences, Faculty of Medicine and Health Sciences, 26656 Ghent University, Ghent 9000, Belgium; 3 Armauer Hansen Research Institute, Addis Ababa 1005, Ethiopia; 4 Department of Microbiology, Immunology and Parasitology, School of Medicine, Addis Ababa University, Addis Ababa 1165, Ethiopia; 5 Faculty of Medicine, Catholic University of Bukavu, Bukavu 019513, Democratic Republic of the Congo; 6 Department of Obstetrics and Gynecology, Hôpital Provincial Général de Référence de Bukavu, Bukavu 019513, Democratic Republic of the Congo; 7 Department of Internal Medicine and Pediatrics, Faculty of Medicine and Health Sciences, 26656 Ghent University, Ghent 9000, Belgium; 8 Departamento de Microbiología y Parasitología, Facultad de Farmacia, 16734 Universidad Complutense de Madrid, Madrid 28040, Spain; 9 Institució Catalana de Recerca I Estudis Avancats (ICREA), Barcelona 08010, Spain

## Abstract

In line with the global research priorities on sexually transmitted infections (STIs), we developed a molecular point-of-care test (POCT) for the parasite that causes the STI trichomoniasis. Trichomoniasis remains the most common curable nonviral STI. We report on the use of specific single-stranded-tailed DNA primers in combination with recombinase polymerase amplification (RPA) in a protein-free nucleic acid lateral flow (NALF) device for use at the point of care. The use of aminated DNA probes eliminates the need for proteins (such as streptavidin or hapten-specific antibodies) for detection of the DNA amplicons, simplifying the manufacturing process and improving reproducibility and cost-effectiveness. The estimated shelf life of the NALF devices is at least 6.6 months at 22 °C, and the devices exhibited high reproducibility. The RPA-NALF requires three simple operator steps with minimal instrumentation and takes approximately 30 min from sample preparation to interpretation of the result. It is specific to and can detect 1.3 × 10^3^ cells/mL with visual readout or 282 cells/mL with the aid of a portable LFA reader. Analysis of the two sets of clinical genomic DNA extracts showed that RPA-NALF is positive for all samples positive by culture assay and for samples with Cq ≤ 23 with the S-DiaMGTV qPCR. Finally, RPA-NALF is positive on biobanked vaginal swabs with Cq ≤ 25 with the Allplex qPCR assays. These results demonstrate that RPA-NALF can specifically detect a moderate load of even in the presence of other microbial DNA and cells and other components of clinical samples.

## Graphical abstract

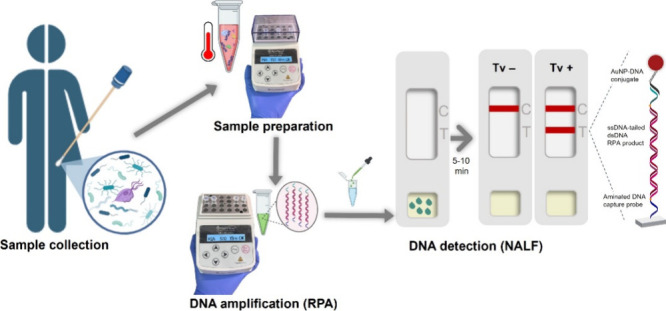

## Introduction

Developing alternative low-cost, rapid point-of-care tests (POCTs) for detecting the sexually transmitted infection (STI) trichomoniasis has been identified as one of the global priority research areas for STIs by the World Health Organization (WHO).^1^ Albeit a nonreportable and a neglected parasitic infection,^2,3^ trichomoniasis remains the most common curable nonviral STI, with 156.3 million cases among adults aged 15–46 years recorded in 2020.^4^ Most cases of trichomoniasis are asymptomatic, but it is associated with adverse pregnancy outcomes,^5,6^ a higher risk of pelvic inflammatory disease and infection with herpes simplex virus type 2 and human immunodeficiency virus.^7^ The global burden of having trichomoniasis was 287,000 disability-adjusted life-years in 2019.^8^ Diagnostic improvements would aid in a better understanding of its public health significance and the creation of better testing guidelines, management strategies, and policies for trichomoniasis and other STIs. A recent review of direct-to-consumer STI testing in the United States indicated that the median cost for testing trichomoniasis is 79 $ for self-collection kits and 109 $ for kits requiring a health care professional for sample collection.^9^

Our group recently developed a molecular detection assay for , the causative agent of trichomoniasis.^10^ In the assay, single-stranded DNA (ssDNA)-tailed amplicons were generated by the isothermal recombinase polymerase amplification (RPA) reaction using ssDNA-tailed primers and detected on a microplate through an enzyme-linked oligonucleotide assay (ELONA) in a sandwich DNA–DNA hybridization format. With the aim to translate this laboratory assay to a molecular POCT format for the detection of , we coupled the tailed-primer-based RPA with a protein-free nucleic acid lateral flow (NALF). There are several studies combining RPA and lateral flow assay (LFA) that are described in detailed reviews.^11–14^ These tests rely on the use of nucleases and/or antibody hapten binding, typically using the TwistDx TwistAmp nfo kit paired with universal LFA devices such as Milenia HybriDetect-1, U-Star units, and PCRD. These LFA devices are designed to detect biotin and FITC/FAM-labeled amplicons, thus relying on the use of streptavidin and hapten-specific antibodies for binding and detection of the amplicons. There are a few reports of RPA-LFA using ssDNA-tailed primers in RPA;^15–21^ however, the LFA strips employed still relied on hapten-labeled complementary DNA.

Herein, protein-free NALF was developed and applied for the detection of RPA amplicons flanked by two ssDNA tails. Protein-free NALFs, first reported in 2007,^22^ provide a more stable, cost-effective, and consistent alternative to the commercial and widely reported systems that rely on protein (antibody)–hapten binding. In this work, RPA was performed for the amplification of a repetitive DNA fragment using ssDNA-tailed primers, leading to the generation of ssDNA-tailed amplicons. This ssDNA-tailed RPA amplicon was then dispensed on the NALF, where one of the tails hybridized with a complementary ssDNA probe linked to gold nanoparticles (DNA-AuNP). As the complex flowed through the strip, the other tail hybridized with a complementary ssDNA probe immobilized via ultraviolet (UV) cross-linking on the nitrocellulose membrane and led to a visual readout (red line), indicating the presence of . Several NALF parameters were optimized, including the composition of the running buffer, the conditions used for the immobilization of the ssDNA probe on the nitrocellulose membrane, and the type of the membrane, as well as the amount of the reporter DNA-AuNP conjugate. Optimized conditions were employed for the final design and application of the test to the analysis of biobanked clinical genomic DNA extracts and clinical vaginal swabs to demonstrate its potential compatibility with patient samples.

## Experimental Section

### Materials

All DNA oligonucleotides (Table SI-1) were purchased from Biomers (Germany). Molecular-biology-grade agarose, Gene Ruler DNA ladder, sodium citrate, gold­(III) chloride trihydrate (HAuCl), Trizma base, IGEPAL CA-630, bovine serum albumin (BSA), and metal-enhanced DAB substrate kit were purchased from Fisher Scientific (Spain). Phosphate-buffered saline (PBS; 10 mM phosphate, 137 mM NaCl, 2.7 mM KCl, pH 7.4), skimmed milk powder, 3,3′,5,5′-tetramethylbenzidine (TMB), EMPIGEN BB, boric acid, tris­(2-carboxyethyl)­phosphine (TCEP), and horseradish peroxidase were purchased from Sigma (Spain). Sodium acetate, acetic acid, sodium chloride, and ethylenediaminetetraacetic acid (EDTA) were purchased from Scharlau (Spain). The TwistAmp basic kit was from TwistDX (United Kingdom), and the GelRed nucleic acid gel stain was from Biotium (Spain).

The buffers used included 10 mM sodium borate buffer, pH 8, 10 mM sodium phosphate buffer, pH 7.4, 10 mM Tris buffer, pH 7.4, and conjugate buffer (5 mM sodium borate buffer, pH 8.8, supplemented with 1% w/v BSA and 10% w/v sucrose). All solutions were prepared by using ultrapure water.

The lateral flow strip material was composed of the cotton linter absorbent pad (grade CF7), nitrocellulose membranes (FF120HP and FF170HP), and the cellulose sample pad (C083), which were purchased from GE Healthcare Life Sciences (Germany), glass fiber (grade 8951) from Ahlmstrom (Finland), and the backing pad from DCN DX (USA). The biodegradable cassettes were purchased from Okos Diagnostics (Netherlands). The CubePlus Portable Lateral Flow Reader (4 cm × 4 cm × 4 cm) from opTricon GmbH (Germany) was used for the measurement of the intensities of the test lines of the NALF.

### Cell Lysates

Crude total cell lysates of *in vitro* cultured microbial isolates were used as DNA templates for RPA. The isolate PH401 was obtained from the PARADET research group of the Universidad Complutense de Madrid, Spain. The following vaginal microbial strains representing common vaginal microbial species were provided by the Laboratory Bacteriology Research (LBR) (Ghent University, Belgium): IHEM 03243, IHEM 04210 and IHEM 04222, CCUG 38953^T^, LMG 11041^T^, UGent 06.41^T^, UGent 21.28 , GS 10234, UGent 09.07, ATCC 700603, LMG 0479^T^, LMG 9203^T^, FB 123-CNA-4, LMG 6414^T^, ATCC 43069, FWO BV 0847, and LMG 14694^T^. Each strain was suspended in PBS with 1% v/v IGEPAL CA-630 at a defined cell concentration and lysed by heating at 95 °C for 3 min. The crude cell lysates containing released DNA were stored at −20 °C until use.

### Biobanked Clinical Genomic DNA

Residual biobanked clinical genomic DNA samples were sourced from the AVEONS study^23^ and from the PARADET group. The AVEONS study was ethically approved by the Internal Review Board of the Catholic University of Bukavu (reference number UCB/CIE/NC/016/2016), by the Ministry of Public Health (reference number 062/CD/DPS/SK/2017), and by the Ethical Committee of Ghent University Hospital (reference number PA2014/003). An informed consent form was signed by each woman who agreed to participate in the study. Genomic DNA was extracted using the RNeasy PowerMicrobiome Kit (Qiagen), following the manufacturer’s instructions omitting the DNase I step. The samples were confirmed positive for using the commercial S-DiaMGTV qPCR assay kit (Diagenode) according to the manufacturer’s instructions for the LightCycler 480 platform.

For the genomic DNA samples from the PARADET group, isolates from vaginal swabs were sourced from a previous study.^24^ Ethical approval was obtained from the Ethics Committee for Research of the Hospital Universitario Puerta de Hierro (Acta no. 21.17). DNA was extracted from these samples using an enzymatic DNA extraction protocol^25^ and the SpeedTools DNA Extraction Kit (Biotools, Spain), whereas the presence of was confirmed by culture assay.^24^

### Biobanked Clinical Vaginal Swab

Residual eluates of clinical vaginal swabs in Amies medium (Eswab, Copan, Italy) were sourced from the IMPRESS study. Ethical clearance and approval was obtained from the Armauer Hansen Research Institute (AHRI)/All Africa Leprosy Rehabilitation and Training Center (ALERT) Ethics Review Committee (Approval No.: PO-04-23), The Ethics Committee of University Ghent and Ghent University Hospital (ONZ 2023 0202), the Departmental Research Ethics Review Committee of the Department of Microbiology, Immunology & Parasitology (DRERC/008/2023), the Institutional Review Board of the College of Health Sciences (053/23/DMIP11/2023), and the Addis Ababa University and Ethical Clearance Committee of Addis Ababa Health Bureau (A/A/11083/227). An informed consent form was signed by each woman who agreed to participate in the study. Clinical vaginal swabs from pregnant women using Eswab (Copan, Italy) were stored at −20 °C in Addis Ababa and then transported to LBR, Ghent, for laboratory analysis. DNA extraction and qPCR were carried out using the Microlab Starlet machine (Hamilton, USA) using the STARMag DNA extraction kit (Seegene Inc., South Korea) and the Allplex STI Essential assay and Allplex Vaginitis screening assay (Seegene Inc., South Korea), respectively.

### Recombinase Polymerase Amplification (RPA)

The RPA reaction mixture using the TwistAmp Basic kit (TwistDx, United Kingdom) was prepared following the RPA conditions optimized in our previous work, combining RPA with the tailed primers and microplate-based colorimetric assay. Briefly, each pellet was reconstituted with a mixture containing 1× rehydration buffer, 240 nM each of the tailed primers, 18 mM magnesium acetate, and nuclease-free water to reach a final reaction volume of 50 μL, including the template DNA (2.5 μL) or nuclease-free water for the no template control (NTC) reaction. Each reaction tube was incubated at 37 °C for 30 min, followed by 2 min at 95 °C for heat termination. Gel electrophoresis for 30 min at 100 V using 2.6% (w/v) agarose and GelRed nucleic acid gel stain in 1× TBE buffer was carried out as a preliminary assessment of the RPA reactions.

### Preparation of the AuNP–DNA Conjugate

Citrate-capped gold nanoparticles (AuNPs) with an approximate size of 20 nm were prepared by the citrate reduction method as previously described.^26^ The absorption spectra of the gold suspension were measured by using the Cary 100 Bio UV–visible spectrophotometer (Agilent). The AuNP–DNA conjugate was prepared using salt aging as previously reported^16^ with minor modifications. Briefly, the thiolated reporter probe DNA (20 μL of 100 μM, Table SI-1) was reduced via addition of 1 μL of 10 mM TCEP and 2 μL of 500 mM acetate buffer, pH 5.2, and letting it incubate for 1 h with mild agitation before adding to 1 mL of the AuNP suspension (optical density (OD) 1). After overnight mixing in dark conditions, a solution consisting of 100 μL of 1 M NaCl and 10 μL of 500 mM Tris–acetate buffer, pH 5.2, was slowly added to the AuNP–DNA mixture at a rate of 10 μL every 20 min and again incubated overnight in dark conditions with mixing. BSA at a final concentration of 1% w/v was then added to the AuNP–DNA mixture and again incubated for 30 min with mild agitation. The AuNP–DNA conjugate was subsequently centrifuged at 15,000 rpm at 10 °C for 30 min, washed three times, and resuspended in 50 μL of conjugate buffer. The concentration and absorption spectra of the functionalized AuNPs were measured (Cary 100 Bio UV–visible spectrophotometer, Agilent). The prepared conjugate was adjusted with conjugate buffer to OD 20 and stored at 4 °C until use.

### Assembly of the NALF Device

The test strip consisted of four overlapping pads, namely, the absorbent pad, detection pad, conjugate pad, and sample pad, assembled on an adhesive backing pad. Specifically, the cotton linter CF7 (Whatman, Germany) served as the absorbent pad, the FF120HP or FF170HP nitrocellulose membranes (Whatman, Germany) as the detection pad, glass fiber (grade 8951, Ahlstrom, Finland) as the conjugate pad, and C083 cellulose fiber (Millipore) as the sample pad. The detection pad was prepared by immobilizing the aminated capture probes via UV cross-linking to the nitrocellulose membrane. The ALFRD automated lateral flow reagent dispenser (Claremont BioSolutions, USA) was used for printing the control line capture probe (CL; 0.7 pmol/mm) and the test line capture probe (TL; 7 pmol/mm). Once the probes were deposited on the membrane, they were exposed to 254 nm UV light at 9 mJ/cm^2^ for 5 min (CL-1000 UV cross-linker, Analytik Jena). The optimum conditions for UV cross-linking were determined by testing a range of UV energy (4.5, 9, or 90 mJ/cm^2^ for 5 min) and exposure times (9 mJ/cm^2^ UV for 2, 5, or 10 min). Additionally, the concentrations of the capture probes were determined by comparing 7 to 0.7 pmol/mm of the control capture probe (CL) and from 13 to 3 pmol/mm of the test capture probe (TL).

The conjugate pad composed of glass fiber (Ahlstrom grade 8951) was pretreated in buffer consisting of 5 mM sodium borate buffer, pH 8.8, with 1% w/v BSA and 0.05% w/v Tween-20 and then dried at 37 °C for at least 2 h. Subsequently, the conjugate pad was immersed in the AuNP–DNA conjugate suspension in conjugate buffer (OD 20) and dried again at 37 °C for at least 2 h before integration in the NALF pad. The amount of AuNP–DNA conjugate dried on the pretreated conjugate pad was OD 10 in the initial experiments, whereas the final amount was determined by comparing OD 10, 15, and 20.

The assembled NALF pad was cut into 3 mm × 6 mm strips (Advanced Sensor Systems P., Ltd.), placed inside the housing cassette, and then stored in a desiccant-sealed bag at ambient environmental conditions until use. Use of the NALF strip or NALF device (strip placed inside the housing cassette) were compared.

### Nucleic Acid Lateral Flow (NALF) Test

Following the RPA reaction, the RPA mixture was diluted 1/20 in running buffer, and 100 μL of the diluted mixture was dispensed on the NALF sample window. In the initial trials using DNA/lysate as template in the RPA reaction, different base running buffers were evaluated, including 10 mM sodium phosphate buffer, pH 7.4, 10 mM sodium borate buffer, pH 8, or 10 mM Tris buffer pH, 7.5.^27^ Moreover, addition of different concentrations of sodium chloride (25, 50, or 100 mM NaCl) to the chosen base running buffer was assessed. The NALF results were monitored at different time intervals (5, 10, 15, and 30 min) to determine the optimum duration of the NALF. The development of two red lines indicates a positive result, a single red upper line indicates a negative result, and a single red lower line or no lines indicate an invalid test.

For the final optimizations of RPA-NALF, lysates of and the common vaginal bacterium were used in the RPA reactions. The RPA reaction mixture was prepared as described previously, with RPA incubation at 37 °C performed for 15, 20, 25, or 30 min. Subsequently, 100 μL of the RPA reactions diluted 1/10 or 1/20 with running buffer (10 mM sodium borate buffer, pH 8, with 50 mM NaCl) was dispensed on the NALF sample window, and the result was checked after 10 min.

### Analytical Specificity and Sensitivity of the RPA-NALF

RPA was carried out as before at 37 °C but now using the optimized duration of 15 min. After amplification, the reaction mixture was diluted 1/20 in 10 mM sodium borate buffer, pH 8, containing 50 mM sodium chloride. Following mixing, a 100 μL aliquot was dispensed on the NALF sample window and the result was recorded after 10 min. For the evaluation of the specificity of the platform, lysates of common vaginal microbial species at 10^6^–10^8^ cell/mL were used as a template in the RPA reaction mixture. NALF was carried out in duplicates, and an image was taken 10 min after dispensing the diluted RPA mixture on the sample window.

To test the analytical sensitivity of the assay, lysates of serial dilutions of cells from 2 × 10^7^ to 2 cells/mL were used. An additional analytical experiment using serial dilutions of synthetic dsDNA was also carried out to determine the assay′s detection limit for pure target DNA. NALF was carried out in duplicate. Following 10 min of NALF runtime, the tests were imaged with a smartphone, and the intensities of the test lines were measured using the CubePlus LFA reader (opTricon GmbH, Germany). The line intensities were analyzed using the GraphPad Prism 8 where a sigmoidal four-parameter logistic (4PL) model was used for curve fitting, and the limit of detection (LOD) was interpolated from the sum of the bottom best-fit value added to three times the standard error of the bottom best-fit value.

### Reproducibility and Stability of the RPA-NALF

RPA-NALF was carried out as described above but using 5 pg/μL synthetic dsDNA as the RPA template for positive reactions and nuclease-free water for the negative reactions. The reproducibility of the assay was evaluated by testing on four different days using different batches of NALF and RPA reactions. For the Arrhenius accelerated thermal stability,^28–31^ assembled NALFs were packed in bags with desiccants and stored at 45 °C for 18 days. RPA was carried out on every testing day of the stored NALFs. Duplicate NALFs were tested. Images were taken after 10 min of runtime, and the Arrhenius equation was used to estimate the storage stability of the NALF device.

### Analysis of Biobanked Clinical Samples

A preliminary application of RPA-NALF with clinical samples was carried out using biobanked clinical genomic DNA and biobanked clinical vaginal swabs. The clinical genomic DNA was used directly as an RPA template while an aliquot of the vaginal swab eluate was heated at 95 °C for 3 min prior to use in the RPA reaction. RPA-NALF was carried out following final conditions similar to the above. Using a portable heater (FastGene Mini Dry Bath), the RPA reactions were incubated at 37 °C for 15 min for amplification followed by heating at 95 °C for 2 min to terminate the reactions. Subsequently, the RPA reactions were diluted 20 times with 10 mM sodium borate buffer, pH 8, containing 50 mM sodium chloride and 100 μL was dispensed on the NALF sample window. The results were recorded after 10 min running time. NALF was carried out in duplicates, and the intensities of the test lines were measured using the CubePlus LFA reader (opTricon GmbH, Germany).

## Results and Discussion

### Design of the RPA-NALF

We report herein the development and evaluation of an RPA-NALF assay (Figure 1) for the detection of the parasite . It requires approximately 30 min and three steps from sample preparation to test result. In this assay, ssDNA-tailed amplicons of DNA are generated after performing RPA with specifically designed ssDNA-tailed primers at 37 °C, which are in turn detected on paper with a NALF strip that does not require hapten–antibody and/or biotin–streptavidin interactions. A clear positive result is produced from a three-part DNA sandwich complex formed when one end of the ssDNA-tailed dsDNA amplicon is hybridized with the nitrocellulose-bound complementary ssDNA probe, and the other end is hybridized with the ssDNA–gold nanoparticle conjugate that generates the red color. Protein-free NALFs are particularly attractive because of the advantages they offer compared to the protein-based ones. The protein-free NALF developed in this work relies on aminated DNA probes for direct covalent immobilization on the nitrocellulose membrane via UV-cross-linking and a thiolated probe for chemisorption on the gold nanoparticles for detection of the DNA amplicon via hybridization. It is proposed as a valid alternative to the commercially available (e.g., Milenia HybriDetect-1, U-Star units, PCRD) and widely reported NALF strips, which utilize proteins such as streptavidin and antibodies for the immobilization of DNA capture probes on the nitrocellulose membrane^32^ and/or capture and detection of biotinylated/hapten-labeled DNAs. With protein-free strips, the manufacturing process is simpler, more reproducible and more cost-effective compared to protein-based strips.^33–35^ The lower cost, associated with both the cost of reagents and the cold chain during transport, is a very important aspect for trichomoniasis testing, considering the lack of affordable and accessible POCTs. The absence of proteins from the strip might also reduce nonspecific signals produced from DNA contaminations of commercial proteins that are not necessarily purified from nucleic acids, and these can interact nonspecifically with the DNA probes.^36^ Protein-free NALF devices are also expected to be more stable during storage because of the absence of temperature-sensitive and chemical-sensitive proteins. We previously reported on the development of a protein-free aptamer lateral flow assay (ALFA) for the detection of and demonstrated that it was stable for at least 1 year at 22 °C.^37^ UV cross-linking as an alternative method to physical and biotin/streptavidin-mediated immobilization of probes on nitrocellulose membranes is also anticipated to lead to a more homogeneous probe immobilization, thus improving the LFA consistency.^38,39^ Moreover, considering that the DNA probes used for strip development are target-independent, the NALF devices are universal and can be used with any target DNA, only requiring the incorporation of the corresponding ssDNA tails into the target primers.

**Figure 1:**
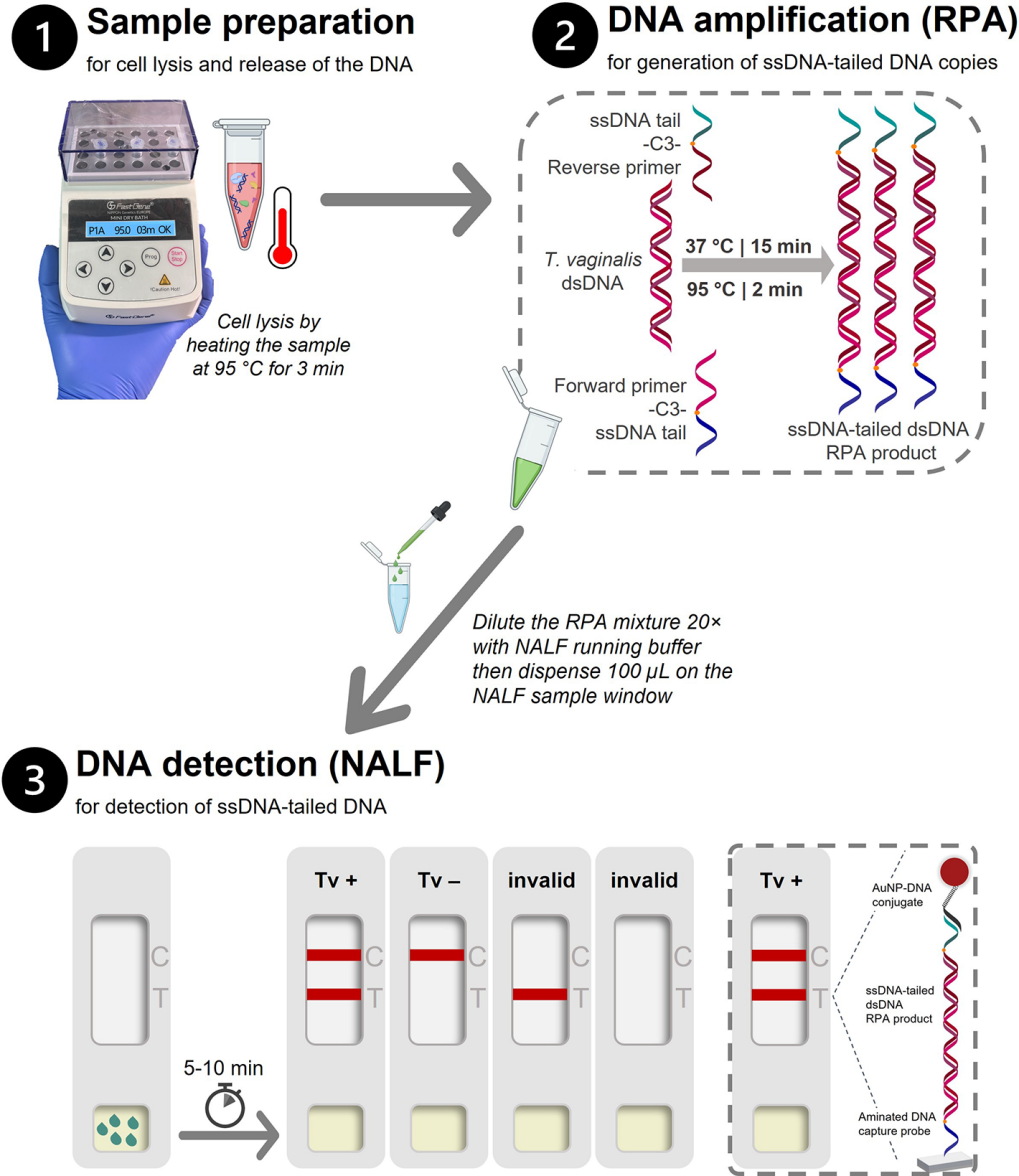
Schematic representation of RPA-NALF developed for the detection of . The assay requires three operator steps and approximately 30 min to complete from sample lysis to result with the following steps: (1) heating at 95 °C for 3 min for cell lysis, (2) RPA at 37 °C for 15 min followed by heat termination at 95 °C for 2 min, and (3) dispensing 100 μL of the 20× diluted RPA mixture on the NALF sample window and reading of result after 5–10 min wherein two lines indicate a positive test for , a single upper line (CL) indicates a valid negative test, and a single lower line (TL) or no line indicate an invalid test. Legend: C, control line; NALF, nucleic acid lateral flow device; RPA, recombinase polymerase amplification; dsDNA, double-stranded DNA; ssDNA, single-stranded DNA; T, test line; Tv, .

### Preparation and Optimization of the NALF Strips and Assay Conditions

The design of the protein-free NALF strips and the conditions employed for sample analysis were optimized to achieve specific detection of . Different common base buffers were first evaluated as the diluents of the RPA reaction. A 10 mM sodium borate buffer, pH 8, was chosen as both the sodium phosphate and Tris buffers resulted in false positive results (Figure SI-1a). Subsequently, a comparison of different UV cross-linking conditions in terms of energy density and duration demonstrated that the highest signal-to-noise was observed when the aminated capture probes were covalently linked to the nitrocellulose membrane by UV 254 nm irradiation at 9 mJ/cm^2^ for 5 min (Figure 2a). A clear test line was also observed in the NALF strip that was not irradiated due to passive adsorption, but UV cross-linking was employed due to the more defined, reproducible, and stable immobilization of the aminated capture ssDNA on the membrane.^40^ The capture probes used in this work have a primary amine group, and a polyT spacer at their 3′-end as this type of modification has been reported to improve the anchoring of DNA on the nitrocellulose membrane and hybridization with complementary DNA.^41,42^ Evaluation of different amounts of the capture probes on the FF120HP nitrocellulose membrane showed that 0.7 pmol/mm of the control capture probe (on CL) (Figure 2b) and 7 pmol/mm of the test capture probe (on TL) (Figure 2c) were optimum for sensitive and specific detection of DNA. The amount of the probe on the CL that hybridizes with the DNA–AuNP conjugate was kept low to balance the CL and TL intensities in the presence of a high amount of analyte and facilitate visual detection of the test result. The probe on the TL that hybridizes with the other ssDNA tail of the RPA product was found to be optimum at 7 pmol/mm since higher or lower amounts did not improve the TL intensity in high and low amounts of cells.

**Figure 2:**
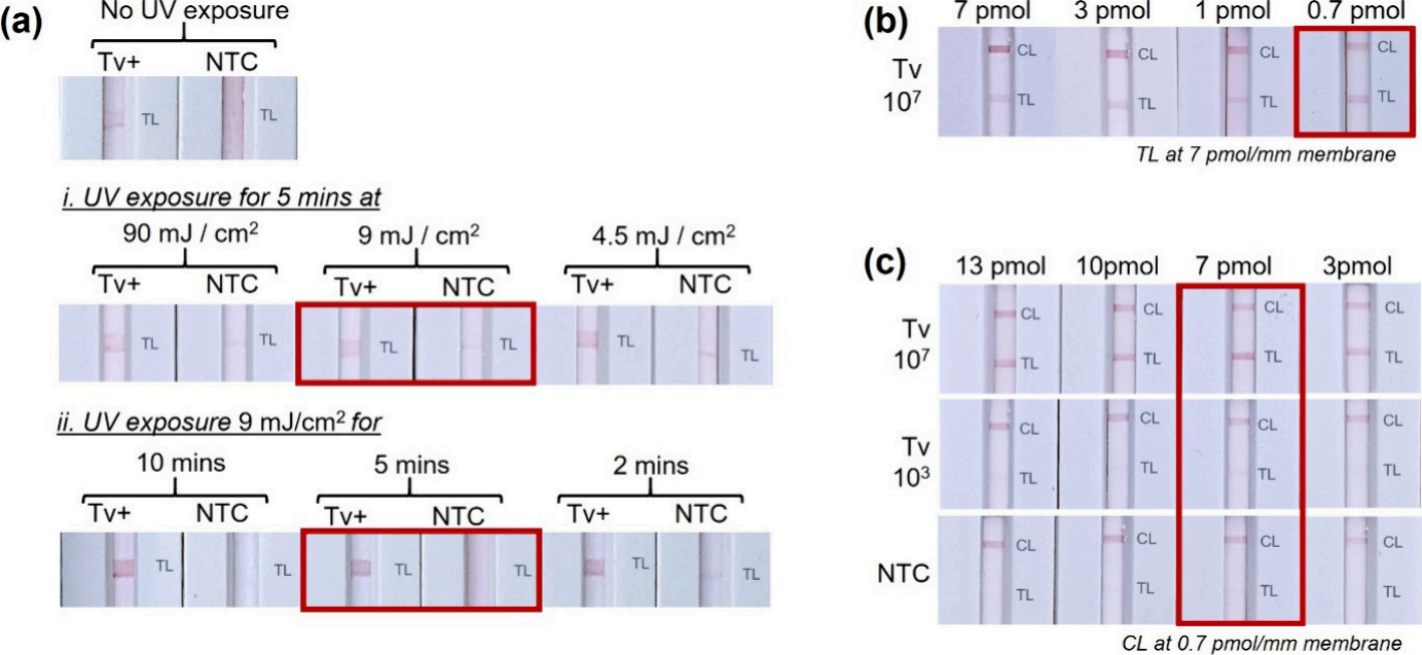
Preparation of the NALF detection pad. (a) UV cross-linking conditions for the immobilization of the ssDNA probes on the membrane, (b) amount of control line capture probe (CL) per millimeter (width) of membrane, and (c) amount of test line capture probe (TL) per millimeter (width) of membrane. Tv+ refers to the use of 10^7^ cells/mL crude lysate for RPA, whereas in (b) and (c), 10^7^ and 10^3^ cells/mL were used. Red boxes highlight the optimal parameters. All RPA reactions were diluted 1/10 prior to NALF analysis. Legend: CL, control line; NTC, no template control; OD, optical density; TL, test line; and UV, ultraviolet.

Regarding the reporter conjugate, bare citrate-capped AuNPs with an approximate size of 20 nm were synthesized based on the UV peak absorbance of 520 nm and TEM images. A shift in the UV maximum peak absorbance was observed after functionalization with the thiolated reporter ssDNA probe, indicative of successful conjugate preparation (Figure SI-2a). The analysis of RPA reactions performed with high (10^7^ cells/mL) and low (10^3^ cells/mL) concentrations of the cells on strips prepared using nitrocellulose membranes with different capillary flow times and different amounts of the AuNP–DNA conjugate showed that OD 20 of the AuNP–DNA conjugate in combination with the FF120HP membrane should be used for the best performance of the assay (Figure SI-2b). A false positive TL was observed with the use of the FF170HP nitrocellulose membrane that has a slower flow rate (140–200 s per 4 cm) than the FF120HP one (90–150 s per 4 cm flow rate).

The NALF conditions optimized thus far facilitated the specific detection of cells, eliminating the generation of false positive results. Aiming at further improving the visual assessment assay, the chosen running buffer (10 mM sodium borate buffer, pH 8) was supplemented with sodium chloride to improve DNA–DNA hybridization by reducing electrostatic repulsion between the negatively charged complementary DNA strands. As can be seen in Figure SI-1b, the intensity of the lines on the membrane was enhanced with increasing concentration of sodium chloride, suggesting improved DNA hybridization. A running buffer composed of 10 mM sodium borate buffer, pH 8, containing 50 mM sodium chloride and 10 min maximum running time was observed to be optimal.

Considering the intended application of the RPA-NALF for the analysis of vaginal swab samples, was tested alongside to represent the nontarget microorganisms commonly found in the vaginal microenvironment. The RPA reaction was performed at 37 °C for 15–30 min, and 1/10 or 1/20 dilutions with the running buffer were performed prior to NALF analysis. As can be seen in Figure 3a, a 15 min RPA reaction time, with a reaction mix diluted 1/20 in running buffer, resulted in the optimum specificity of the RPA-NALF. Finally, the importance of housing the NALF strip inside a cassette was confirmed by comparing use of the NALF device and NALF strip in assessing the RPA reactions. The result was more specific with the NALF device (strip housed inside the cassette) than with the NALF strip (Figure 3b), which can be attributed to better controlled fluid flow along the strip. Furthermore, evaluation of NALF over a series of running times demonstrated that the NALF result should be read at a maximum of 10 min after sample addition. For research laboratory-scale production and use, the cost of a single RPA-NALF test, exclusive of manpower and facilities, is estimated to be 1.82 € with 0.80 € for a 10 μL RPA reaction mixture and 1.02 € for the NALF device with the running buffer (Table SI-2).

**Figure 3:**
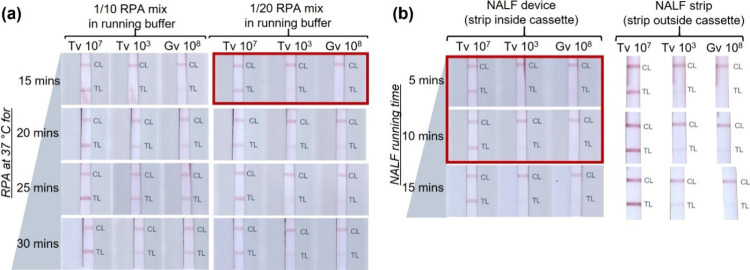
Optimization of executing the RPA and NALF steps. (a) Duration of the RPA amplification step and dilution of the RPA reaction mix in the NALF running buffer. The RPA reaction mixture was incubated at 37 °C for 15–30 min and then diluted 1/10 or 1/20 in running buffer (10 mM sodium borate buffer, pH 8, with 50 mM sodium chloride) and left to run for 10 min. (b) Duration of NALF and comparison of using the NALF strip and NALF device. The RPA reaction mixture was incubated at 37 °C for 15 min and then diluted 1/20 in running buffer and left to run for 5–15 min. Shown are the RPA-NALF results for the target (Tv at 10^7^ cells/mL and 10^3^ cells/mL) and the nontarget (Gv at 10^8^ cells/mL).

### Specificity and Sensitivity of the RPA-NALF

Subsequently, the optimized RPA-NALF was tested against a panel of common vaginal microorganisms to evaluate its specificity. A positive result based on the development of two lines on the NALF strip was observed only with , demonstrating that the presence of the other microorganisms in the sample did not interfere with the assay (Figure 4 and Table SI-3).

**Figure 4:**
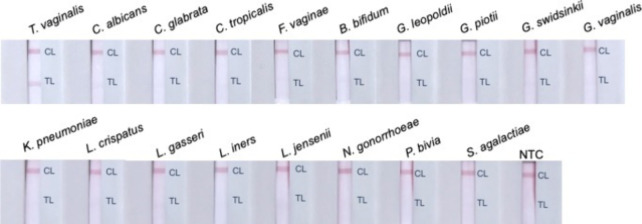
Specificity of RPA-NALF. Representative strips from duplicate tests are shown. Legend: CL, control line; TL, test line; and NTC, no template control.

To assess the sensitivity of RPA-NALF, serially diluted cells were lysed and analyzed using optimized conditions. As can be seen in Figure 5, the developed assay had a visual detection limit of 1.3 × 10^3^ cells/mL. When a portable lateral flow reader was used to measure the test line intensities, a sigmoidal curve was fitted to the data and the detection limit was calculated to be 282 cells/mL. The developed RPA-NALF assay has comparable sensitivity to the commercial antigen-based immunochromatographic OSOM test that has a detection limit of at least 2.5 × 10^3^ cells/mL.^43^ It is less sensitive though than the reported RPA-based CRISPR-Cas12a assay system combined with the lateral flow strip that can detect 10 cells/mL using purified genomic DNA^44^ and the PCR-based POCT Visby Medical Sexual Health Test that can detect 0.24–1.2 cells/mL.^45^ However, the CRISPR-Cas12a system requires multiple steps and relatively expensive enzymes, and the Visby PCR test is inherently laboratory-based and costly. Different commercial PCR-based and published isothermal nucleic acid amplification tests (NAATs) for are summarized in Table SI-4. Even though further work is required to improve the sensitivity of the RPA-NALF, its simplicity, based on few operator steps and no requirement for expensive laboratory infrastructure, simply a portable heater, its rapid execution (DNA sample preparation to NALF completion in approximately 30 min), and facile visual readout are highly attractive features for a POCT.

**Figure 5:**
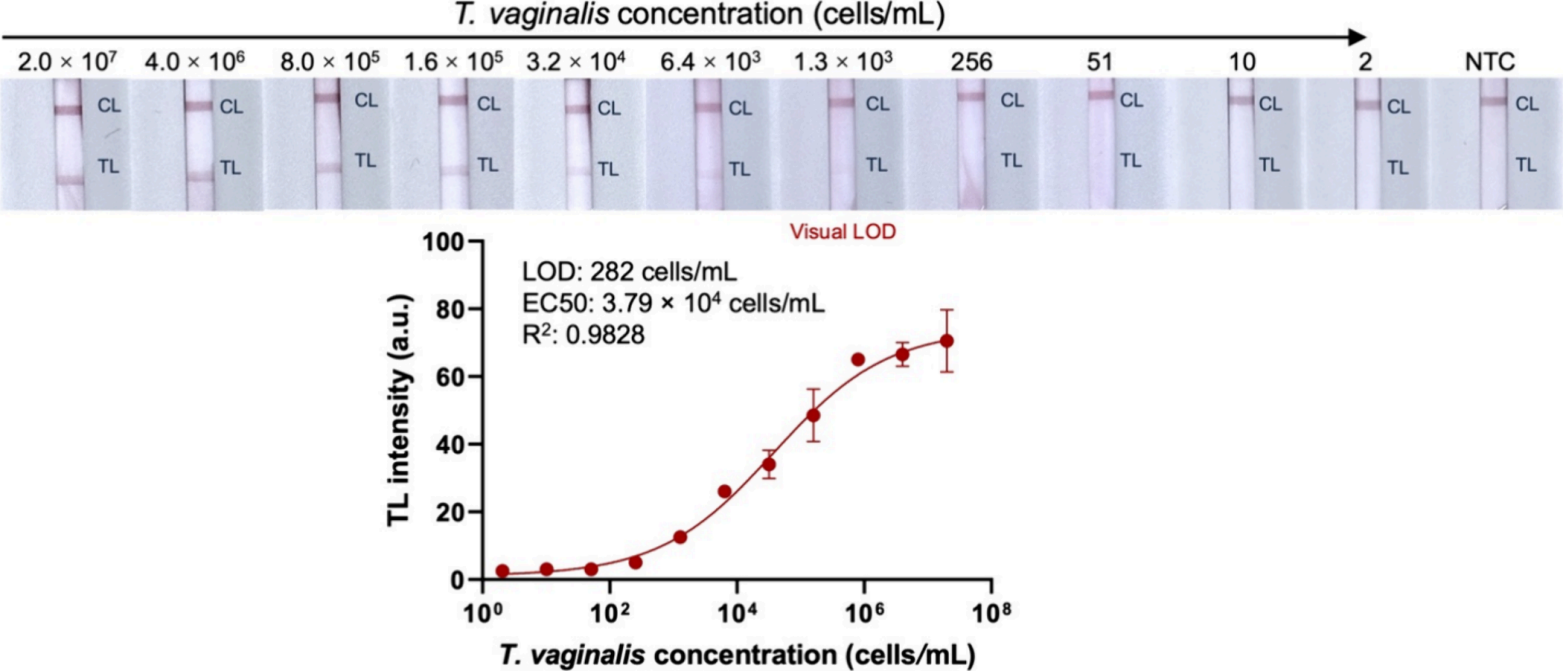
Calibration curve of the RPA-NALF assay using crude lysates of serially diluted cells. The test line intensities were quantified using the CubePlus LFA reader (opTricon GmbH, Germany). Error bars represent NALF strips from duplicate tests. Legend: au, arbitrary units; EC50, half maximal effective concentration; LOD, limit of detection; *R* ^2^: coefficient of determination.

### Reproducibility and Stability of the RPA-NALF

Beyond specificity and sensitivity, other key aspects of RPA-NALF are reproducibility and stability. To eliminate any potential variability associated with cultured cells, synthetic dsDNA (Figure SI-3) was used as the template in RPA. To assess reproducibility, the assay was performed on four separate days. No significant differences were observed in the intensities of the test lines when dsDNA was used as template in the RPA reactions, and no nonspecific signals appeared in the absence of the target DNA (Figure SI-4), demonstrating that the assay is reproducible. The stability of the NALF devices was evaluated through an accelerated thermal stability study.^42–45^ After the devices were stored for 18 days at 45 °C, the test lines retained approximately the same signal intensity as in day 0 (Figure SI-5), suggesting no significant loss of stability. Based on the Arrhenius equation, the estimated storage stability of the NALF devices is at least 6.6 months at 22 °C. While the accelerated thermal stability study gave an initial estimate of the stability of this protein-free LFA device, a real-time, long-term stability study under varying temperature and humidity conditions is needed to provide more accurate data and identify any potential weaknesses, ultimately ensuring a consistent and reliable assay.

### Detection of in Biobanked Clinical Samples

A preliminary diagnostic evaluation of the effectiveness of the proposed RPA-NALF assay to detect in clinical samples was finally performed using a total of 43 biobanked clinical genomic DNA samples and 10 biobanked clinical vaginal swab samples. The total DNA extracts were prepared as detailed in the experimental section and analyzed with RPA-NALF under optimized conditions. Analysis of the RPA reactions by gel electrophoresis was also conducted for each set of clinical samples previously tested for by culture assay (Figure SI-6) and by qPCR using the S-DiaMGTV kit (Figure SI-7) and the Allplex STI Essential and Allplex Vaginitis screening assays (Figure SI-8). A 100% agreement between RPA-NALF and the traditional culture assay was observed for the set of 22 -positive samples (Figure 6).

**Figure 6:**
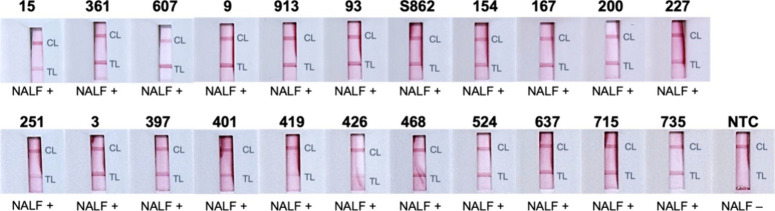
RPA-NALF analysis of biobanked genomic DNA samples previously tested positive for using the culture assay. Sample identification in bold font. The interpretation of the RPA-NALF results is shown as positive (+) or negative (−) for . Legend: CL, control line; NTC, no template control; TL, test line.

In another set of 21 samples previously analyzed by qPCR using the S-DiaMGTV qPCR kit, positive RPA-NALF was observed in eight samples with a Cq span of approximately 15–23 (Figure 7a). Negative RPA-NALF was observed in 13 samples with Cq > 25, with some found to harbor DNA of other STI agents such as , , and . Correlation analysis indicated strong and significant correlation (*r*_pb_ = 0.8448, *p* < 0.0001) between the qPCR Cq values and the RPA-NALF result (Figure 7b). These results indicate that the RPA-NALF can detect a moderate load of in clinical samples, while the presence of other microbial DNA, expected in a clinical genitourinary specimen, does not interfere in the developed test.

**Figure 7:**
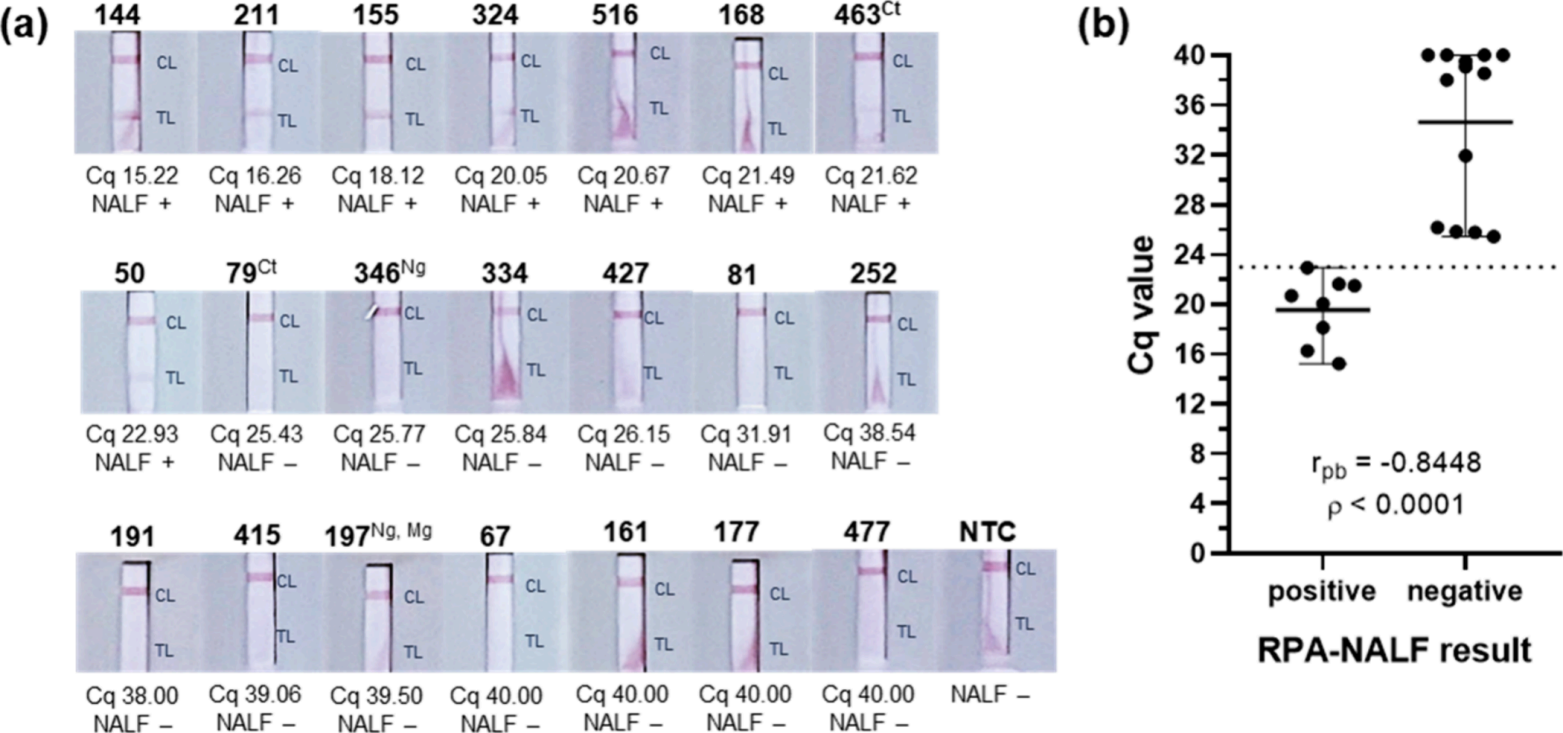
RPA-NALF analysis of biobanked genomic DNA samples previously tested for using the S-DiaMGTV qPCR kit (Diagenode). (a) Image of NALF with the sample identification in bold font and superscript indicating other STI agent detected in the same sample by another qPCR assay. (b) Correlation between qPCR Cq values and RPA-NALF result. qPCR was used for detection of . The interpretation of the RPA-NALF results is shown as positive (+) or negative (−) for . Legend: CL, control line; TL, test line; Cq, qPCR quantitative cycle; Ct, ; Mg, ; Ng, ; NTC, no template control; *r*_pb_, point-biserial correlation; *p*, significance value.

Finally, using 10 clinical vaginal swab eluates, we demonstrated the suitability of the RPA-NALF assay in detecting in complex clinical samples by achieving a perfect correlation between RPA-NALF and qPCR (Table 1 and Figure SI-9). We were able to detect moderate loads of with Cq ≤ 25 when a different qPCR kit was used as the reference (Allplex STI Essential assay and Allplex Vaginitis screening assay). The analysis of these samples also demonstrated the high specificity of the RPA-NALF to even in the presence of other STI agents and vaginitis-associated microorganisms at varying abundances (Table SI-5), and other components of vaginal swabs eluted in Amies medium. Additional information on the qPCR-based detection of STI and vaginitis-associated microorganisms in the swab samples is provided in Table SI-5.

**Table 1:** RPA-NALF Analysis of Biobanked Vaginal Swab Samples Previously Tested in qPCR Using the Allplex STI Essential Assay and Allplex Vaginitis Screening Assay^a^

		qPCR (Seegene Allplex)			
		TV			
sample ID	TV RPA-NALF result	result	Cq	comorbidity	bacterial vaginosis interpretation
117	positive	positive	13.77	Uu, Mh, Up, Gv, Av, Mob	bacterial vaginosis
377	positive	positive	14.94	Uu, Mh, Gv, Av, Mob	bacterial vaginosis
420	positive	positive	24.61	Up, Lacto, Mob	normal
215	negative	negative	n.d.	Up, Ct, Ca, Lacto, Gv, Av, Mob	normal
440	negative	negative	n.d.	Up, Lacto, Gv, Mob	normal
387	negative	negative	n.d.		normal
197	negative	negative	n.d.	Uu, Mg, Ca, Lacto, Gv, Av, Mob	intermediate
348	negative	negative	n.d.	Uu, Mh, Up, Lacto, Ca,Gv, Av, Mob	intermediate
298	negative	negative	n.d.	Ca, Co, Gv, Av, Mob	bacterial vaginosis
321	negative	negative	n.d.	Up, Ca, Gv, Av, Mob	bacterial vaginosis

a Legend: Tv, ; Uu, ; Ng, ; Mh, ; Mg, ; Up, ; Ct, ; Ca, s; Co, *Candida* others; Lacto, *Lactobacillus* spp.; Gv, ; Av, ; Mob, *Mobiluncus* spp.; BV, bacterial vaginosis; n.d., not detected.

## Conclusion and Future Directions

A molecular POCT for the detection of was developed. The assay exploits ssDNA-tailed primers for the isothermal amplification of genomic DNA and straightforward colorimetric detection of the amplified DNA by DNA–DNA hybridization on paper using a protein-free NALF device. The independence of this device from proteins to mediate DNA amplicon detection simplifies manufacturing, making it more reproducible and cost-effective and with higher expected stability than protein-dependent devices. Based on a short-term accelerated thermal stability study, a storage stability of at least 6.6 months at 22 °C was demonstrated, but long-term real time studies are required for more accurate data to ensure reliable assay results under varying conditions. The developed RPA-NALF is low-cost, simple, and rapid, with the assay costing less than 2 €, which is significantly less expensive than current TV tests, and the cost of the developed test with a scaling perspective is anticipated to be significantly lower. Testing for using the developed assay required three steps and approximately 30 min from DNA sample preparation to visual readout of positive or negative result in NALF. The small footprint, the programming capabilities and the low energy requirements of the portable heater used in this work for thermal lysis of the samples and for RPA ensure the compatibility of the assay with POCT.

The assay showed to be specific to and could detect as low as 282 cells/mL with the aid of a portable LFA reader or 1.3 × 10^3^ cells/mL by visual inspection. Evaluation with biobanked clinical samples demonstrated that the assay could detect moderate load of , and it does not suffer any interference from DNA of other STI agents. The assay had perfect concordance with the culture assay and strong correlation with qPCR Cq values, with a positive RPA-NALF result in biobanked genomic DNA samples with Cq ≤ 23. Finally, the assay was also able to detect in a small set of biobanked vaginal swab samples with Cq ≤ 25 containing other relevant vaginal microorganisms, confirming its suitability for detecting in complex clinical samples.

Ongoing work is focused on testing a larger number of diverse clinical samples, including those with comorbidities, to ensure reliable and sensitive results across different stages of infection. The product profile for trichomoniasis POCT set by the WHO will also serve as a guide in future optimizations and validation of the assay.

## Supplementary Material



## Notes

Oligonucleotides used in this study (Table SI-1); estimated cost of laboratory scale production of RPA-NALF (Table SI-2); RPA-NALF against and common vaginal microbial isolates (Table SI-3); commercial PCR-based and published isothermal NAATs for (Table SI-4); analysis of clinical vaginal swab samples by qPCR using the Allplex STI Essential assay and Allplex Vaginitis screening assay (Table SI-5); NALF running buffer base and salt supplement (Figure SI-1); preparation of the AuNP–DNA conjugate and optimization of the amount used for the NALF (Figure SI-2); calibration curve of RPA-NALF assay using serially diluted synthetic dsDNA (Figure SI-3); reproducibility of the RPA-NALF assay (Figure SI-4); accelerated stability study of the RPA-NALF assay (Figure SI-5); analysis of biobanked clinical genomic DNA samples by RPA-gel electrophoresis with culture assay as the reference assay for detection (Figure SI-6); analysis of biobanked clinical genomic DNA samples by RPA-gel electrophoresis with qPCR as the reference assay (Figure SI-7); analysis of biobanked clinical vaginal swab samples by RPA-gel electrophoresis with qPCR as the reference assay (Figure SI-8); analysis of biobanked clinical vaginal swab samples with the RPA-NALF (Figure SI-9); and Arrhenius accelerated thermal stability study (PDF)
