# Supplementary material for: Development of a Protein-Free Nucleic Acid Lateral Flow Assay for
Source: Anal Chem. 2025 Jul 8;97(28):15093–103. doi: 10.1021/acs.analchem.5c01006 (PMC12291036; doi:10.1021/acs.analchem.5c01006)
Supplement: Supplementary file 1 [file ac5c01006_si_001.pdf]

SUPPLEMENTARY INFORMATION

Development of a protein-free nucleic acid lateral flow assay for *Trichomonas vaginalis*

Christine Aubrey C. Justo,[1] Miriam Jauset-Rubio,[1] Vasso Skouridou,[1*] Piet Cools,[2] Lisa Himschoot,[2] Abel Abera Negash,[3,4] Guy Mulinganya Mulumeoderhwa, [5-7] Alexandra Ibáñez-Escribano,[8] and Ciara K. O´Sullivan[1,9*]

[1] Interfibio Consolidated Research Group, Departament d'Enginyeria Química, Universitat Rovira i Virgili, 43007 Tarragona, Spain

[2] Department of Diagnostic Sciences, Faculty of Medicine and Health Sciences, Ghent University, 9000 Ghent, Belgium

[3] Armauer Hansen Research Institute, 1005 Addis Ababa, Ethiopia

[4] Department of Microbiology, Immunology and Parasitology, School of Medicine,

Addis Ababa University, 1165 Addis Ababa, Ethiopia

[5] Faculty of Medicine, Catholic University of Bukavu, 019513 Bukavu, Democratic Republic of the Congo

[6] Department of Obstetrics and Gynecology, Hôpital Provincial Général de Référence de Bukavu, 019513 Bukavu, Democratic Republic of the Congo

[7] Department of Internal Medicine and Pediatrics, Faculty of Medicine and Health Sciences, Ghent University, 9000 Ghent, Belgium

[8] Departamento de Microbiología y Parasitología, Facultad de Farmacia, Universidad Complutense de Madrid, 28040 Madrid, Spain

[9] Institució Catalana de Recerca I Estudis Avancats (ICREA), 08010 Barcelona, Spain

*Corresponding authors: ciara.osullivan@urv.cat, vasoula.skouridou@urv.cat

Index

Table SI-1. Oligonucleotides used in this study……………………..………………..……S3

Table SI-2. Estimated cost of laboratory scale production of RPA-NALF…………………………………………………..…………………………S4

Table SI-3. RPA-NALF against *T. vaginalis* and common vaginal microbial isolates..…S5

Table SI-4. Commercial PCR-based and published isothermal NAATs for *T. vaginalis*……………………………………………………………….………………S6

Table SI-5. Analysis of clinical vaginal swab samples by qPCR using the Allplex™ STI Essential assay and Vaginitis screening assay…………………… ……………..…S8

Figure SI-1. NALF running buffer base and salt supplement.…….……………………..….S9

Figure SI-2. Preparation of the AuNP-DNA conjugate and optimization of the amount used for the NALF.………….……………………………………….……………...S10

Figure SI-3. Calibration curve of RPA-NALF assay using serially diluted synthetic *T. vaginalis* dsDNA……….…………...…………………………………………S11

Figure SI-4. Reproducibility of the RPA-NALF assay.…………………………………..…S12

Figure SI-5. Accelerated stability study of the RPA-NALF assay.……………….………….…...S13

Figure SI-6. Analysis of biobanked clinical genomic DNA samples by RPA-gel electrophoresis with culture assay as the reference assay for *T. vaginalis* detection...S14

Figure SI-7. Analysis of biobanked clinical genomic DNA samples by RPA-gel electrophoresis with qPCR as the reference assay……………………………………S15

Figure SI-8. Analysis of biobanked clinical vaginal swab samples by RPA-gel electrophoresis with qPCR as the reference assay……………………………………S16

Figure SI-9. NALF image of of biobanked clinical vaginal swab samples taken after 10 minutes of dispensing the diluted RPA mixture…………………………………S17

Arrhenius accelerated thermal stability study…………………………………………….S18

References……………………………………………………………………………………S19

Table SI-1. Oligonucleotides used in this study.

Name	Sequence (5' – 3')
Tailed forward primer	AACCTTAGAGCGGATTAGGG-C3-TTCTTCAACAACAGTAGATATTGCTTGCGACGGG
Tailed reverse primer	TGTAAAACGACGGCCAGT-C3-ATGAAAACTTCTGGAGCATATTGGTATCCG
NALF control line capture probe (CL)	TGTAAAACGACGGCCAGT-T_{15}- C6-amine
NALF test line capture probe (TL)	CCCTAATCCGCTCTAAGGTT-T_{15}- C6-amine
NALF reporter probe	ACTGGCCGTCGTTTTACA-T_{15}-C3-thiol

Table SI-2. Estimated cost of laboratory scale production of RPA-NALF

**Item**	**Cost per unit (€)**
RPA reaction mix (10 µL)	0.8087
NALF device	
Cassette (*no shipping fee)	0.2000
Backing pad	0.0112
Absorbent pad	0.0041
Sample pad	0.0010
Detection pad	0.1950
Conjugate pad	0.6012
Running buffer (190 µL)	0.0035
**TOTAL**	**1.8247**

Table SI-3. RPA-NALF against *T. vaginalis* and common vaginal microbial isolates

Group	Strains	Concentration (cells/mL)	RPA-NALF result
Protozoa	*T. vaginalis* PH401	2.0×10^7	positive
Yeast	*Candida albicans* IHEM 03243	6.7×10^6	negative
	C. glabrata IHEM 04210	6.7×10^6	negative
	C. tropicalis IHEM 04222	6.7×10^6	negative
Bacteria	*Fannyhessea vaginae* CCUG 38953^T	1.5×10^8	negative
	Bifidobacterium bifidum LMG 11041^T	1.5×10^8	negative
	Gardnerella leopoldii UGent 06.41^T	1.0×10^8	negative
	G. piotii UGent 06.41^T	7.2×10^7	negative
	G. swidsinskii GS 10234	6.0×10^7	negative
	G. vaginalis UGent 09.07	1.5×10^8	negative
	Klebsiella pneumoniae ATCC 700603	1.5×10^8	negative
	Lactobacillus crispatus LMG 0479^T	1.5×10^8	negative
	L. gasseri LMG 9203^T	1.5×10^8	negative
	L. iners FB123-CNA-4	1.5×10^8	negative
	L. jensenii LMG 6414^T	1.5×10^8	negative
	Neisseria gonorrhoeae ATCC 43069	1.5×10^8	negative
	Prevotella bivia FWO BV 0847	1.5×10^8	negative
	Streptococcus agalactiae LMG 14694^T	1.5×10^8	negative

Table SI-4. Commercial PCR-based and published isothermal NAATs for *T. vaginalis* (TV)

Method	*T. vaginalis* detection limit	Analyte	Ref. #
Commercial test kits (PCR-based DNA amplification)			
Visby Medical Sexual Health Test	1.2 TV/mL	TV ATCC 30001, metronidazole susceptible	1
	0.24 TV/mL	TV ATCC 30238, metronidazole resistant	
S-DiaMGTV ™ qPCR kit	1000 copies/mL	genomic DNA (Vircell); kit control	2,3
Aptima *Trichomonas vaginalis* Assay (Panther)	0.003 TV/mL	TV in negative clinical liquid pap specimens collected in PreservCyt solution	4
Aptima *Trichomonas vaginalis* Assay (Tigris)	0.01 TV/mL		
BD MAX™ CT/GC/TV assay; all-in-one extraction and real-time PCR for the simultaneous detection of CT, GC, and TV	5-10 cells/mL	TV in vaginal swab	5
	10-34 cells/mL	TV in urine	
XPERT® TV assay, cartridge-based Real-time PCR	2 cells/mL	TV-spiked vaginal swab matrix	6
	3 cells/mL	TV-spiked urine	
STD Direct Flow Chip Kit, multiplex PCR+colorimetric immunoenzymatic system	10 copies/reaction	synthetic DNA	7
Published assays (isothermal DNA amplification)			
LAMP assay	1 trichomonad and 1 pg of TV DNA/reaction	genomic DNA	8
	10 cells/reaction	genomic DNA	9

	1 cell/mL	genomic DNA from spiked genital swabs	10
	1 cell/mL	genomic DNA from spiked urine samples	
	0.036 ng/μL	genomic DNA	11
	100 cells/mL	genomic DNA	
Thermophilic helicase-dependent amplification (assay with lateral flow immunoassay readout	~ 7 genomic equivalents of TV DNA/mL of sample	clinical urine	12
	0.25 genomic equivalents of TV DNA/mL	genomic DNA in buffered urine	
RPA-based CRISPR-Cas12a assay system combined with lateral flow strip	10 cells/mL	genomic DNA	13
Multi-enzyme isothermal rapid amplification-CRISPR/Cas13a-lateral flow device	0.01 fg/μL	plasmid DNA	14
	1 pg/μL	genomic DNA	
RPA-NALF	1.3×10^3 Tv/mL	Genomic DNA (heat-lysed Tv in buffer)	This study
	282 Tv/mL		

Table SI-5. Analysis of clinical vaginal swab samples by qPCR using the Allplex™ STI Essential assay and Allplex™ Vaginitis screening assay. Legend: Tv, *Trichomonas vaginalis*; Uu, *Ureaplasma urealyticum*; Ng, *Neisseria gonorrhoeae*; Mh, *Mycoplasma hominis*; Mg, *Mycoplasma genitalium*; Up, *Ureaplasma parvum*; Ct, *Chlamydia trachomatis*; Ca, *Candida albicans*; Co, *Candida* others; Lacto, *Lactobacillus* spp.; Gv, *Gardnerella vaginalis*; Av, *Atopobium vaginae*; Mob, *Mobiluncus* spp.; BV, bacterial vaginosis.

Sample ID	RPA-NALF	Present STI and vaginitis-associated microorganisms	BV Interpretation	Cq, Allplex™ STI Essential Assay							Cq, Allplex™ Vaginitis Screening Assay						
				Tv	Uu	Ng	Mh	Mg	Up	Ct	Tv	Ca	Co	Lacto	Gv	Av	Mob
117	Positive	Tv, Uu, Mh, Up, Gv, Av, Mob	Bacterial Vaginosis	13.77	33.48	-	17.8	-	26.65	-	13.62	-	-	-	17.21	17.65	31.7
377	Positive	Tv, Uu, Mh, Gv, Av, Mob	Bacterial Vaginosis	14.94	27.68	-	16.76	-	-	-	13.72	-	-	-	20.27	19.60	35.6
420	Positive	Tv, Up, Lacto, Mob	Normal	24.61	-	-	-	-	24.91	-	22.65	-	-	17.9	-	-	35.43
215	Negative	Up, Ct, Ca, Lacto, Gv, Av, Mob	Normal	-	-	-	-	-	23.6	19.32	-	30.45	-	16.8	25.26	31.56	27.99
440	Negative	Up, Lacto, Gv, Mob	Normal	-	-	-	-	-	23.38	-	-	-	-	18.91	26.68	-	31.85
387	Negative	-	Normal	-	-	-	-	-	-	-	-	-	-	-	-	-	-
197	Negative	Uu, Mg, Ca, Lacto, Gv, Av, Mob	Intermediate	-	23.99	-	-	32.19	-	-	-	26.54	-	12.32	14.45	25.14	28.31
348	Negative	Uu, Mh, Up, Lacto, Gv, Av, Mob	Intermediate	-	28.88	-	25.97	-	23.98	-	-	-	-	10.51	21.86	27.21	23.52
298	Negative	Ca, Co, Gv, Av, Mob	Bacterial Vaginosis	-	-	-	-	-	-	-	-	26.65	29.28	-	11.23	15.27	31.29
321	Negative	Up, Ca, Gv, Av, Mob	Bacterial Vaginosis	-	-	-	-	-	23.06	-	-	34.76	-	-	35.54	24.16	29.69

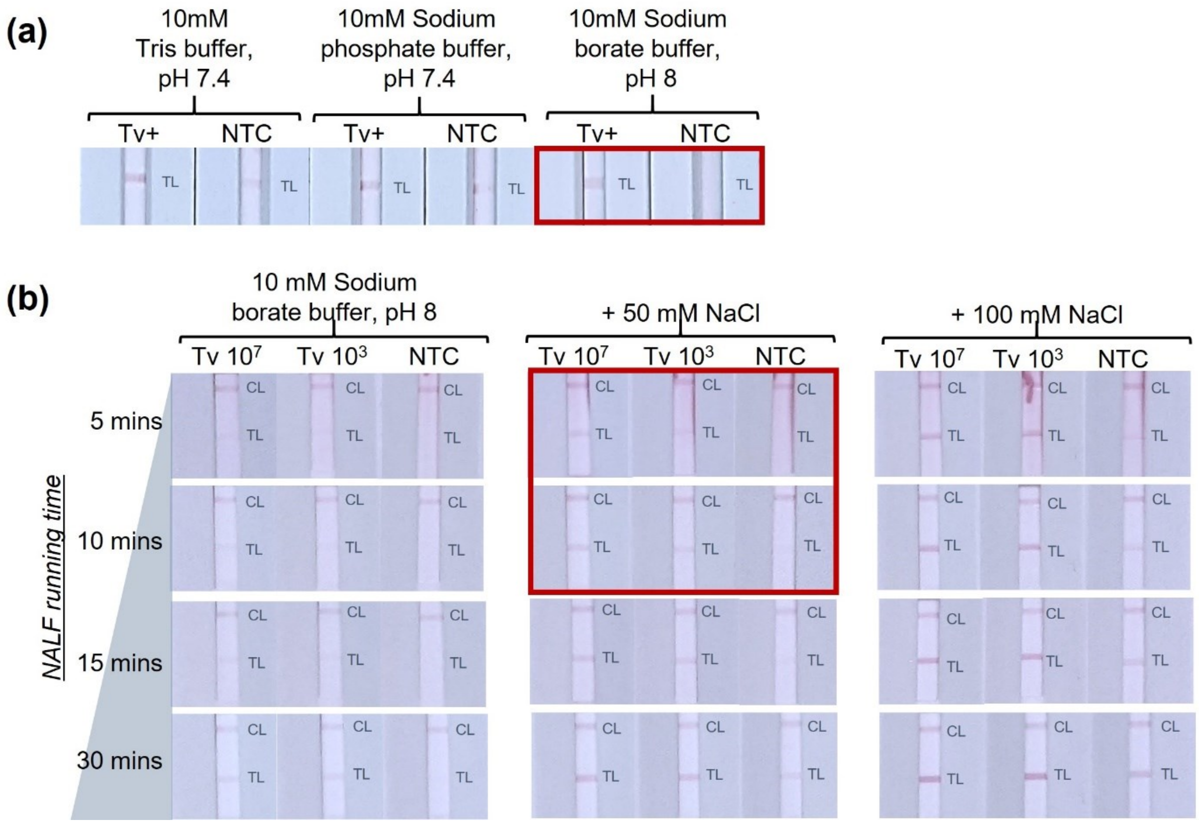

Figure SI-1. NALF running buffer (a) base and (b) salt supplement. Tv+ in (a) refers to the use of 10^7 *T. vaginalis* cells/mL crude lysate for RPA, whereas in (b) 10^7 and 10^3 *T. vaginalis* cells/mL were used. *T. vaginalis* cells (10^7 and 10^3 cells/mL) were used to prepare the crude lysate for RPA. NTC refers to the no-template control RPA reaction. All RPA reactions were diluted 1/10 prior to NALF analysis. Legend: CL, control line; NTC, no template control; TL, test line.

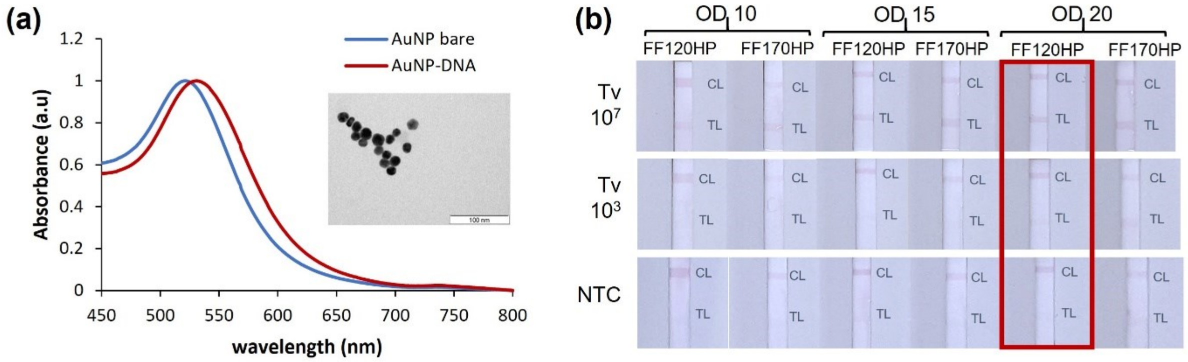

Figure SI-2. Preparation of the AuNP-DNA conjugate and optimization of the amount used for the NALF. (a) Visible spectra of the synthesized citrate-capped AuNPs before and after conjugation with the ssDNA probe. The inset shows a TEM image of the bare AuNPs. (b) Amount of AuNP-DNA conjugate on the conjugate pad and comparison of FF120 and FF170 nitrocellulose membranes. *T. vaginalis* cells (10^7 and 10^3 cells/mL) were used for the preparation of the crude lysate for RPA. All RPA reactions were diluted 1/10 prior to NALF analysis. Legend: a.u., arbitrary unit; CL, control line; NTC, no template control; OD, optical density; TL, test line.

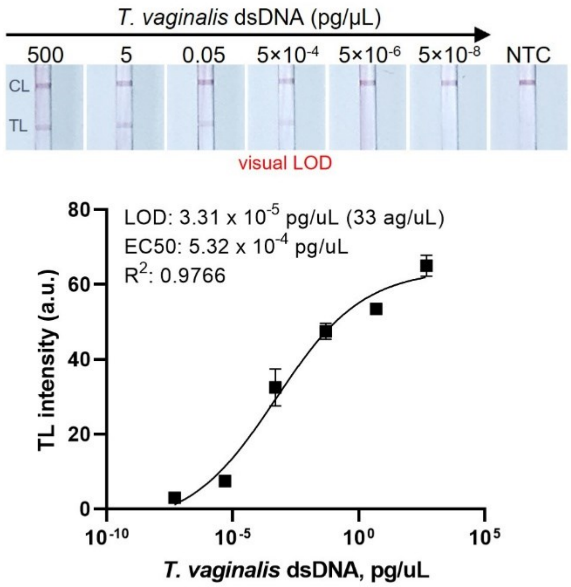

Figure SI-3. Calibration curve of RPA-NALF assay using serially diluted synthetic *T. vaginalis* dsDNA. The test line intensities were quantified using the CubePlus LFA reader (opTricon GmbH, Germany). Error bars represent NALF strips from duplicate tests. Legend: CL, control line; TL, test line; NTC, no-template control RPA reaction; a.u., arbitrary units; EC50, half maximal effective concentration; LOD, limit of detection; R^2: coefficient of determination.

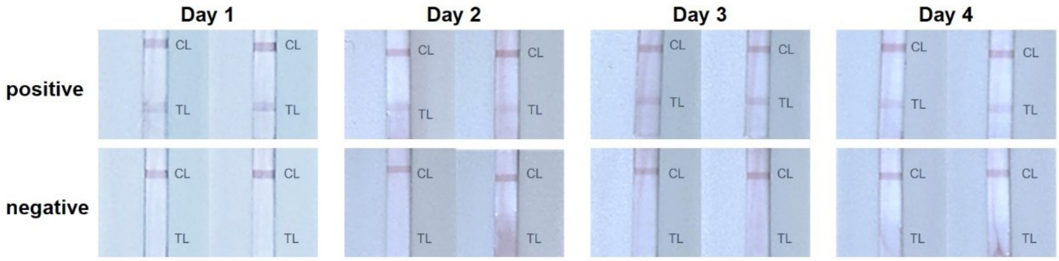

Figure SI-4. Reproducibility of the RPA-NALF assay. The assay was performed on four separate days using synthetic *T. vaginalis* dsDNA (5 pg/µL) as the RPA template in the positive reaction. Nuclease-free water was used as template in the negative reaction. Legend: CL, control line; TL, test line.

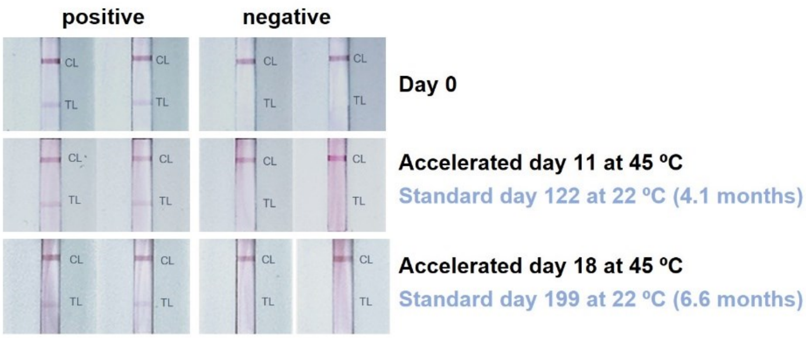

Figure SI-5. Accelerated stability study of the RPA-NALF assay. Detection of synthetic *T. vaginalis* dsDNA with RPA-NALF after storing the NALF devices for 18 days at 45 ºC. The Arrhenius equation was used to estimate the storage stability of the NALF device based on this short accelerated thermal stability study. Synthetic *T. vaginalis* dsDNA (5 pg/µL) served as the RPA template in the positive reaction. Nuclease-free water was used as template in the negative reaction. Legend: CL, control line; TL, test line.

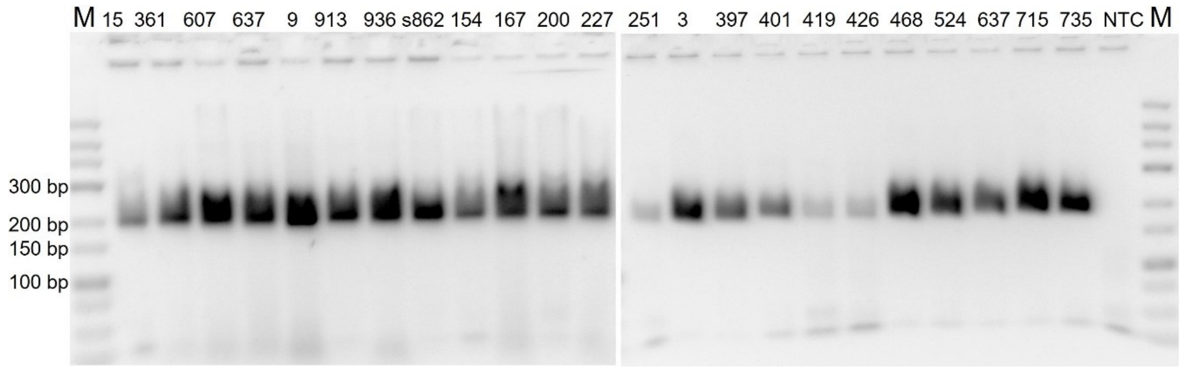

Figure SI-6. Analysis of biobanked clinical genomic DNA samples by RPA-gel electrophoresis with culture assay as the reference assay for *T. vaginalis* detection. Legend: M, DNA marker; NTC, no template control.

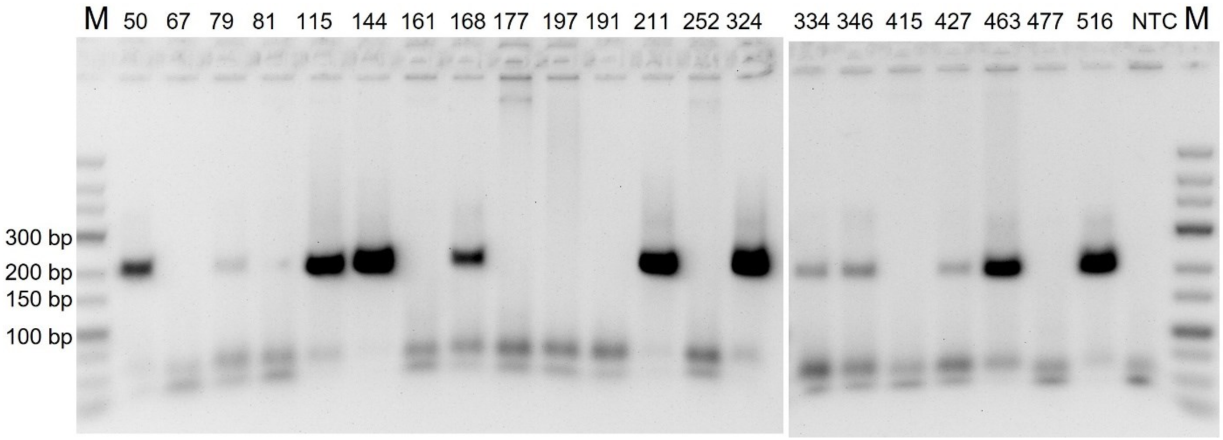

Figure SI-7. Analysis of biobanked clinical genomic DNA samples by RPA-gel electrophoresis with qPCR as the reference assay. *T. vaginalis* and other STI agents were detected by qPCR using the DiaMGTV™ kit (Diagenode). Legend: M, DNA marker; NTC, no template control.

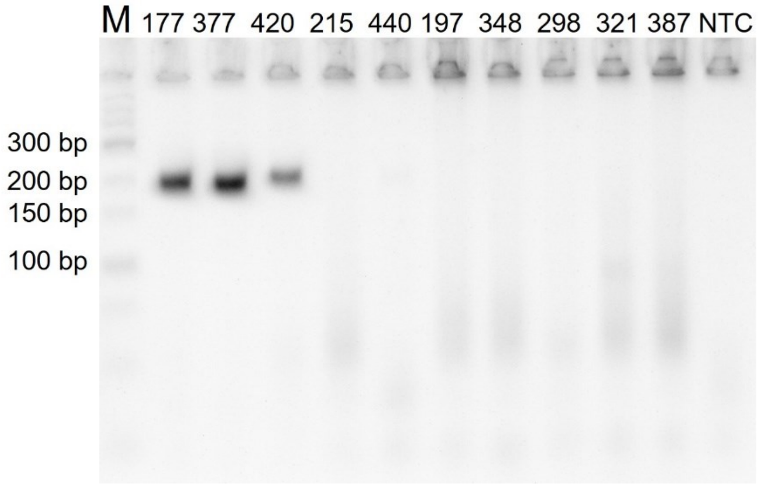

Figure SI-8. Analysis of biobanked clinical vaginal swab samples by RPA-gel electrophoresis with qPCR as the reference assay. *T. vaginalis* and other microorganisms were detected by qPCR using the Seegene Allplex™ STI Essential assay and Vaginitis screeing assay. Legend: M, DNA marker; NTC, no template control.

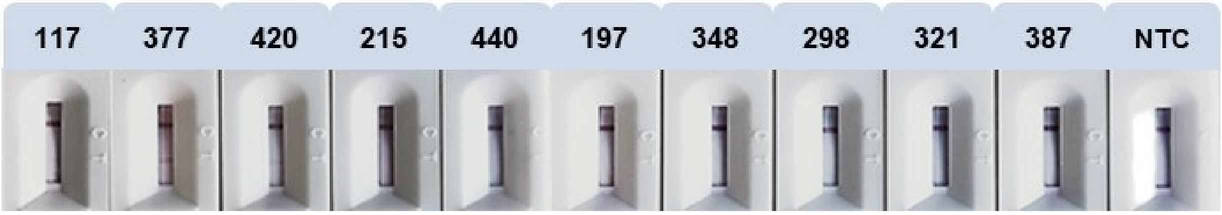

Figure SI-9. Analysis of biobanked clinical vaginal swab samples with the RPA-NALF. Sample IDs are in bold font.

Arrhenius accelerated thermal stability study. The Arrhenius equation (equation 1)[15,16] was used to calculate the rate constants of the degradation process at the different temperatures (equation 2), considering a constant molar activation energy Ea[17]. The storage stability was finally estimated using equation 3.

Equation 1 $k = Ae^{(-\frac{Ea}{RT})}$

Equation 2 $\ln\left(\frac{k_{T1}}{k_{T2}}\right) = \left(\frac{Ea}{R}\right) * (\frac{1}{T2} - \frac{1}{T1})$

Equation 3 $t_{T2} = t_{T1} * (\frac{k_{T1}}{k_{T2}})$

where k is the rate constant of the degradation process at a specific temperature T for the LFA being tested, Ea is the molar activation energy of the process, R the universal gas constant, T the temperature at which the LFA is being tested, A is a pre-exponential factor (a constant), and k_{T1} and k_{T2} are the rate constants of degradation at the temperature the LFA is being stored at for short period of time (T1) and the one for which stability is being predicted (T2).

References

(1) Visby Medical, Visby Medical Sexual Health Test Instruction for Use (2024). https://www.visbymedical.com/assets/sexual-health-test/Visby-Medical-Sexual-Health-Test-Instructions-for-Use.pdf (accessed on 18 July 2024)

(2) Vanmassenhove, B.; Hervent, A.-S.; Persijn, L.; Vynckier, L.; Alliet, G. Analytical Performance of the Multiplex S-DiaMGTV Kit (Diagenode) for the Detection of *Mycoplasma genitalium* and *Trichomonas vaginalis* in Cervical Smear Specimens. Poster presented at the 10th European Meeting on Molecular Diagnostics, Noordwijk aan Zee, Netherlands, October 11-13, 2017. https://www.azoostende.be/sites/default/files/2023-11/Analytical-performance-of-the-multiplex-S-DiaMGTV-kit-Diagenode-for-the-detection-of-MG-and-TV-in-cervical-smear-specimens.pdf

(3) Chesnay, A.; Lancelin, B.; Brun, C. Le; Pastuszka, A.; Desoubeaux, G.; Lanotte, P. Contribution of a molecular test for the diagnosis of genital infection with *Trichomonas vaginalis* and *Mycoplasma genitalium*. *Annales de Biologie Clinique* **2020**, *78* (6), 623–627. DOI: 10.1684/abc.2020.1589

(4) Hologic Inc., 510(k) Summary: Aptima Combo 2 assay (Panther and Tigris Sytem) and Aptima Trichomonas vaginalis assay (Panther and Tigris Sytem). **2020**. https://www.accessdata.fda.gov/cdrh_docs/pdf20/K200436.pdf. (accessed on 18 July 2024)

(5) Kawa, D.; Kostiha, B.; Yu, J. H.; LeJeune, M. *Elevating the standard of care for STIs: the BD MAX CT/GC/TV assay*. **2017**. https://moleculardiagnostics.bd.com/wp-content/uploads/2017/08/CT-GC-TVWhitepaper.pdf (accessed on 18 July 2024)

(6) Cepheid. *Xpert ® TV Instructions for Use*. **2022**. https://www.cepheid.com/content/dam/www-cepheid-com/documents/package-insert-files/Xpert-TV-ENGLISH-Package-Insert-301-2887-Rev.__D.pdf. (accessed on 18 July 2024)

(7) Barrientos-Durán, A.; de Salazar, A.; Alvarez-Estévez, M.; Fuentes-López, A.; Espadafor, B.; Garcia, F. Detection of sexually transmitted disease-causing pathogens from direct clinical specimens with the multiplex PCR-based STD Direct Flow Chip Kit. *European Journal of Clinical Microbiology and Infectious Diseases* **2020**, *39* (2):235-241. DOI: 10.1007/s10096-019-03686-w

(8) Goo, Y. K.; Shin, W. S.; Yang, H. W.; Joo, S. Y.; Song, S. M.; Ryu, J. S.; Kong, H. H.; Lee, W. K.; Chung, D. Il; Hong, Y. Loop-Mediated Isothermal Amplification Targeting Actin DNA of *Trichomonas vaginalis*. *The Korean Journal of Parasitology* **2016**, *54* (3), 329–334. DOI: 10.3347/kjp.2016.54.3.329

(9) Li, Y.; Wang, S.; Li, H.; Song, X.; Zhang, H.; Duan, Y.; Luo, C.; Wang, B.; Ji, S.; Xie, Q.; Zhang, Z. Development of a convenient detection method for *Trichomonas vaginalis* based on loop-mediated isothermal amplification targeting adhesion

protein 65. *BMC Infectious Diseases* **2020**, *20* (1), 319. DOI: 10.1186/s12879-020-05048-w

(10) Reyes, J. C.; Solon, J. A.; Rivera, W. L. Development of a loop-mediated isothermal amplification assay for detection of *Trichomonas vaginalis*. *Diagnostic Microbiology and Infectious Disease* **2014**, *79* (3), 337–341. DOI: 10.1016/j.diagmicrobio.2014.03.016

(11) Adao, D. E. V.; Rivera, W. L. Loop-mediated isothermal amplification (LAMP) assay for the rapid detection of the sexually-transmitted parasite, *Trichomonas vaginalis*. *Annals of Parasitology* **2016**, *62* (1), 25–31. DOI: 10.17420/ap6201.28

(12) Rosenbohm, J. M.; Robson, J. M.; Singh, R.; Lee, R.; Zhang, J. Y.; Klapperich, C. M.; Pollock, N. R.; Cabodi, M. Rapid electrostatic DNA enrichment for sensitive detection of *Trichomonas vaginalis* in clinical urinary samples. *Analytical Methods* **2020**, *12* (8), 1085–1093. DOI: 10.1039/c9ay02478f

(13) Li, S.; Wang, X.; Yu, Y.; Cao, S.; Liu, J.; Zhao, P.; Li, J.; Zhang, X.; Li, X.; Zhang, N.; Sun, M.; Cao, L.; Gong, P. Establishment and application of a CRISPR-Cas12a-based RPA-LFS and fluorescence for the detection of *Trichomonas vaginalis*. *Parasites and Vectors* **2022**, *15* (1), 350. DOI: 10.1186/s13071-022-05475-5

(14) Yang, Z.; Wang, J.; Qi, Y.; Shi, Y.; Li, F.; Wang, W.; Tian, X.; Mei, X.; Zhang, Z.; Wang, S. A novel detection method based on MIRA-CRISPR/Cas13a-LFD targeting the repeated DNA sequence of *Trichomonas vaginalis*. *Parasites and Vectors* **2024**, *17* (1), 1–12. DOI: 10.1186/s13071-023-06106-3

(15) Laboria, N.; Fragoso, A.; O'Sullivan, C. K. Storage Properties of Peroxidase Labeled Antibodies for the Development of Multiplexed Packaged Immunosensors for Cancer Markers. *Analytical Letters* **2011**, *44*(11), 2019–2030. DOI: 10.1080/00032719.2010.539732

(16) Bever, C. S.; Adams, C. A.; Hnasko, R. M.; Cheng, L. W.; Stanker, L. H. Lateral flow immunoassay (LFIA) for the detection of lethal amatoxins from mushrooms. *PLoS One* **2020**, *15* (4), No. e0231781. DOI: 10.1371/journal.pone.0231781

(17) VivaCheck Biotech (Hangzhou) Co., Ltd. Accelerated Stability Study Report for VivaDiag™ SARS-CoV-2 Ag Rapid Test. https://www.mmbiotech.it/wp-content/uploads/2020/09/TF025-003-Accelerated-Stability-Study-Report-for-VivaDiag-SARS-CoV-2-Ag-Rapid-Test.pdf. (accessed on 16 December 2024)
